# Effects of routine procedures on salivary cortisol in mechanically ventilated neonates

**DOI:** 10.1038/s41598-023-29913-1

**Published:** 2023-03-21

**Authors:** Wanting Li, Huiyue Zhang, Xianghui Huang, Ruming Ye, Ying Lin

**Affiliations:** 1 Fujian Provincial key Laboratory of Neonatal Diseases, Xiamen, China; 2 Xiamen Children’s Hospital, Xiamen, China; 3 Jinjiang Municipal Hospital, Jinjiang, China

**Keywords:** Health care, Medical research

## Abstract

Even though the stress secondary to invasive procedures has been investigated, less attention has been paid to the stimulation or pain caused by routine procedures on neonates. The changes in salivary cortisol concentration in mechanically ventilated NICU patients during routine procedures were monitored to provide reference and guidance for pain management. 80 mechanically ventilated neonates in the hospital from Sep 2021 to Mar 2022 were selected. The salivary cortisol levels of the neonates were monitored during nursing procedures and were categorized by their risk levels to the following groups: high-risk (endotracheal suctioning and arterial blood sampling), moderate-risk (gastric tube insertion, venipuncture), and low-risk (bedside bathing and diaper changes). The changes in heart rate were also recorded and compared. The concentration of cortisol in the saliva of the neonates was 1.5 ± 0.8 nmol/L during the sleeping state, 6.2 ± 1.3 nmol/L during endotracheal suctioning, 6.4 ± 1.4 nmol/L during arterial blood sampling, 6.1 ± 1.2 nmol/L during venipuncture, 4.4 ± 1.1 nmol/L during gastric tube insertion, 3.5 ± 0.8 nmol/L during bedside bathing, and 3.3 ± 0.9 nmol/L during a diaper change. The results revealed a statistically significant effect between routine procedures on salivary cortisol levels. Compared with the neonates in the control sleep state, there was a significant (P < 0.05) change in salivary cortisol concentration of infants undergoing high and moderate-risk nursing procedures. There was a small but significant (P < 0.05) change in salivary cortisol levels in infants who underwent low-risk procedures compared to infants in the control sleep state. Further, the fluctuation of salivary cortisol levels in routine procedures was more frequent compared with routine handling at night. The fluctuations of salivary cortisol concentration in high-risk procedures were larger than that of infants who underwent low-risk procedures, with the difference being statistically significant (P < 0.05). It was also determined that the top four influencing factors on the infants’ heart rate were arterial blood sampling venipuncture, intubation, endotracheal suctioning, and gastric tube insertion (P < 0.05). Monitoring the saliva cortisol concentration index and heart rates can reflect the impact of different routine procedures on newborns and can be used to manage neonatal pain in the future.

## Introduction

Mechanical ventilation is a basic rescue technique that can be life-saving but also causes varied physiological and psychological pain to patients. Studies have shown that typically, newborns experience 16 painful stimulations per day during hospital admission^1^. Mechanically ventilated newborns may experience as many as 17.3 painful stimulations per day^2^. Endotracheal intubation is a stressful, painful, and potentially dangerous procedure. Experts in neonatal pain management agree that endotracheal intubation can be classified as moderate to severely painful^3^. Repeated painful stimuli cause several short and long-term adverse effects on newborns^4^. The hypothalamic–pituitary–adrenal axis (HPA axis) is an essential endocrine axis of the human body, with cortisol being its primary end product. The functional state of the HPA axis and cortisol levels reflect the human body’s ability to adapt to a particular environment and respond to stimuli to a certain extent^5^. Cortisol (cortis01), a “stress hormone”, is a glucocorticoid secreted by the adrenal cortex, increasing in concentration on the occurrence of stress, pain, anxiety, or acute tissue injury. Salivary cortisol results from the passive transportation of serum-free cortisol to the saliva through acinar diffusion. It reflects the real-time dynamic changes in the level of serum-free cortisol and is often used as a sensitive index to monitor the degree of psychological stress^6^. Reducing the stimulation or stress of neonates mechanically ventilated in the NICU environment during invasive procedures is a hot topic for research. Even though the stress secondary to invasive procedures has been explored, not enough attention has been paid to whether routine procedures can cause adverse stimulation or pain to neonates. To understand the influence of routine procedures on neonates, this study explored the change of the concentration of salivary cortisol in mechanically ventilated neonates in the NICU environment. This manuscript reports the changes in salivary cortisol levels in neonates during routine procedures such as endotracheal suctioning, arterial blood sampling, bedside bathing, and gastric tube insertion. The reasons for the changes in the salivary cortisol levels were analyzed and recorded as reference and guidance for pain management in neonates.

## Methods

Eighty neonates with neonatal respiratory distress syndrome treated in the hospital from Sep 2021 to Mar 2022 were recruited in the study. All mechanically ventilated neonates were intubated orotracheally, with or without sedation and analgesia. The inclusion criteria were: 1. meeting indications for mechanical ventilation^7^, 2. clinical symptoms of progressive dyspnea with decreasing laboratory pO_2,_ and 3. informed consent from parents or guardians. The exclusion criteria were: 1. the requirement for mechanical ventilation < 24 h, 2. diagnosis of congenital metabolic disorders, 3. congenital malformations, and 4. the presence of consciousness disorders or incomplete clinical data.

Methods: The changes in salivary cortisol levels were observed in neonates according to the risk classification of nursing procedures^8^ after routine high-risk (endotracheal suctioning and arterial blood sampling), moderate-risk (gastric tube insertion, venipuncture) and low-risk procedures (bedside bathing and diaper changing). The clinical staff undertaking the procedure and obtaining salivary samples consisted of 10 nurses with more than 5 years of experience, all of whom have undergone uniform training and assessment. According to the collection time point of each procedure, the data were collected during routine procedures for the same child, referring to related research^9^. The saliva samples of the child were collected by a German SARSTED saliva collection tube for 30 min before and after the procedure. Infants mechanically ventilated had to be sedated, providing a high degree of cooperation. During the sample collection process, a cotton swab was placed in the saliva collector under the child’s tongue for 3 to 5 min^10,11^. Next, the cotton swab was removed and placed into its dedicated sleeve. When the cotton swab could not be kept under the tongue for 3 to 5 min due to improper technique or accidental dislodgement out of the mouth or into the throat, it was replaced immediately, and the process was repeated. Special care was taken not to mix the saliva sample with sputum or blood. The saliva samples were collected and frozen at − 20 °C or centrifuged on the same day or the following day^12^.

### Ethics declarations

This study was performed in accordance with the Declaration of Helsinki, and approved by the Scientific Ethics Committee of Xiamen Children’s Hospital ([2021] No.17). All guardian of participants signed the written informed consent to participate in the study.

### Observation indicators

Monitoring of salivary cortisol: ELISA was used to monitor the salivary cortisol levels. Saliva collection was carried out using specially-designed German SARSTED saliva collection tubes. The samples were centrifuged at 300 r/min for 5 min and stored at − 80 °C^12^. The samples were tested in batches within 6 months. The samples were thawed at room temperature for 30 min and tested with a salivary cortisol immunodiagnostic kit^13^.

Real-time fluctuation of heart rate was noted by observing the value on the ECG monitor 5 min before and after the procedure and recording the heart rate.

### Statistical analysis

The SPSS statistical software package (Version 20.0, IBM Corporation, Armonk, New York, USA) was used for statistical analysis. Data conforming to normal distribution was expressed by ${\overline{\text{x}}}$ ± s. A comparison between groups was made using the paired *T*-test. The original non-normal distribution data were logarithmically converted (y = lgx) and expressed by M(Q1, Q3). If the data was normal distribution after conversion, it was expressed by X-S, and a T-test was performed. Counting data was represented by value (%). The x^2^ test was used for comparison between groups. P < 0.05 was deemed statistically significant.

## Results

### General data of participants

A total of 80 neonates were included in this study, including 44 boys (55%) and 36 girls (45%). The birth weight was (1.87 ± 0.71) kg. The duration of mechanical ventilation time was (7.52 ± 4.74) days, and the gestational age at birth was (28.55 ± 5.96) weeks.

### Comparison of salivary cortisol

**Figure 1:**
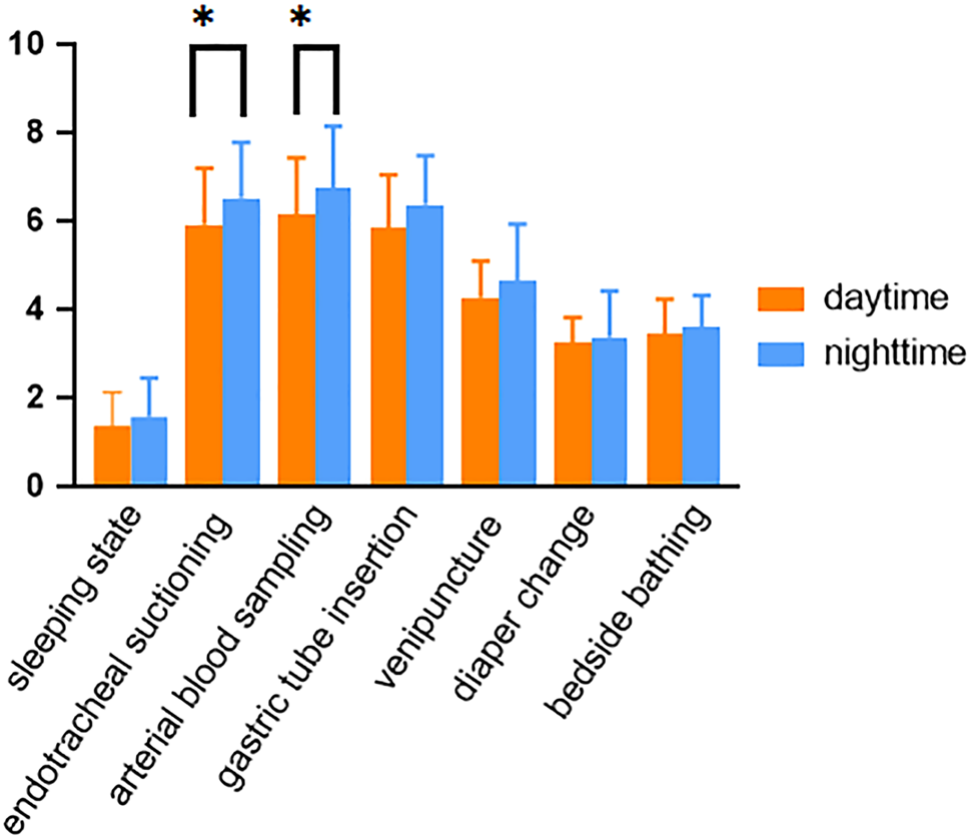
Comparison of daytime and nighttime salivary cortisol in neonates.

The concentration of cortisol in the saliva of the children was 1.5 ± 0.8 nmol/L during the sleeping state, 6.2 ± 1.3 nmol/L during endotracheal suctioning, 6.4 ± 1.4 nmol/L during arterial blood sampling, 6.1 ± 1.2 nmol/L during venipuncture, 4.4 ± 1.1 nmol/L during gastric tube insertion, 3.5 ± 0.8 nmol/L during bedside bathing, and 3.3 ± 0.8),9 nmol/L during a diaper change. The results revealed a statistical difference between groups and a statistically significant effect between routine procedures on salivary cortisol levels. Compared with the children in the sleeping state, the concentration of salivary cortisol in high-risk procedures [endotracheal suctioning (t = 27.6, P < 0.05) and arterial blood sampling(t = 27.8, P < 0.05)], moderate-risk procedures [venipuncture(t = 28.7, P < 0.05) and gastric tube insertion(t = 19.5, P < 0.05)] changed significantly. The concentration of salivary cortisol in low-risk procedures [bedside bathing(t = 16.3, P < 0.05) and diaper change(t = 14.0, P < 0.05)] changed slightly but was still higher than neonates in the sleeping state, the difference being statistically significant, as shown in Table 1. Compared with routine procedures at night, the salivary cortisol in routine daytime procedures fluctuated more frequently. In fact, there were more fluctuations in high-risk procedures compared to low-risk procedures, with the difference being of statistical significance (P < 0.05), as shown in Fig. 1.

**Table 1:** Changes of saliva cortisol concentration in neonates with mechanical ventilation under different routine operations (nmol/L, ${\overline{\text{x}}}$  ± s).

Procedure		Salivary cortisol level	F	*P*
High risk procedures	Endotracheal suctioning	6.2 ± 1.3	186.2	0.00
	Arterial blood sampling	6.4 ± 1.4		
Moderate risk procedures	Gastric tube insertion	6.1 ± 1.2		
	Venipuncture	4.4 ± 1.1		
Low risk procedures	Diaper change	3.3 ± 0.9		
	Bedside bathing	3.5 ± 0.8		
Sleep state		1.5 ± 0.8*		

The collection time of each procedure: endotracheal suctioning (9:00, 21:00); Diaper changes (10:00, 04:00); Venipuncture (at admission); Bedside bathing (8:30); Gastric tube insertion (11:00); Arterial blood sampling (30 min after admission); Sleep state (once every night when sleeping).

### Comparison of heart rate changes in children

The top four influencing factors on the participants’ heart rates were arterial blood sampling, venipuncture and intubation, endotracheal suctioning, and gastric tube insertion (P < 0.05), as shown in Table 2.

**Table 2:** Effects of routine manipulation on heart rate in children (times/min, ${\overline{\text{x}}}$ ± s).

Procedure	Before procedure	After procedure	t	*P*
Endotracheal suctioning	133.0 ± 7.0	162.8 ± 11.3	− 20.1	0.00
Gastric tube insertion	135.3 ± 8.7	163.1 ± 13.2	− 15.7	0.00
Arterial blood sampling	137.1 ± 8.0	167.1 ± 9.7	− 21.4	0.00
Venipuncture	133.9 ± 5.2	150.9 ± 9.6	− 13.9	0.00
Diaper change	134.6 ± 7.1	143.0 ± 8.3	− 6.9	0.00
Bedside bathing	137.9 ± 10.1	146.1 ± 10.1	− 5.2	0.00

## Discussion

Intubation is a stressful, painful, and potentially dangerous procedure. Therefore, techniques to reduce the stimulation or stress of mechanically ventilated neonates in the NICU during invasive procedures are active research topics. There has been extensive research on the stress caused by invasive procedures, but very few studies have reported the influence of routine procedures on stress in neonates. In this study, the stress or pain response of mechanically ventilated neonates after routine procedures was observed. It is well known that cortisol plays a key role in brain development and function^9^ Multiple studies have shown that the cortisol levels of premature infants are also related to repeated painful stimuli during hospitalization and that this effect continues until school age^14,15^. Cortisol, the main glucocorticosteroid of the human body, is synthesized by the fascicular zone cells of the adrenal cortex. The adrenal cortex secretes 15–25 mg of cortisol daily, and the hypothalamus–pituitary–adrenal cortex axis regulates its biosynthesis and secretion. Since the cell membrane of the fascicular zone of the adrenal cortex contains receptors that specifically bind to corticotropin E when stimulated. The synthesis and secretion of corticotropin E occur within 1–2 min. The secretion of corticotropin can be directly stimulated by a corticotropin-releasing hormone from the hypothalamus^12^. The results revealed that both high-risk and low-risk nursing procedures caused irritation or pain in the sleep state, and the cortisol concentration in saliva changed significantly during arterial blood sampling, endotracheal suctioning, venipuncture, and gastric tube insertion. These changes in cortisol concentration suggest that stimuli from routine procedures impacted the neonates and, therefore, it may be beneficial to comfort them during these procedures (Table 1).

Neonatal stress can increase catecholamine levels, causing fluctuations in blood pressure. Especially in premature infants, the increase in catecholamine levels and the corresponding fluctuations in blood pressure are the main causes of intracranial hemorrhage^3^. The incidents of intracranial hemorrhage are highest at 5 to 7 days of life. Reports indicate that patients in the intensive care unit may suffer from pain due to many factors, such as primary disease processes, surgery, trauma, handling, wound care, endotracheal intubation, and insertion and removal of drainage tubes. For example, the discomfort from nursing procedures can develop into chronic ICU-related pain, increasing the functional load on the individual organ systems. This can lead to a stress reaction, lack of sleep or metabolic changes, fatigue, and disorientation, even causing mortality^7^. Reducing the pain of mechanically ventilated newborns and relieving them of stress reaction of mechanically ventilated newborns is of great importance to stabilize the circulation of children and reduce oxygen consumption in mechanical ventilation treatment.

The findings of this study revealed that the concentration of saliva cortisol changed during diaper changes and bedside bathing compared with those in the sleeping state, suggesting that the nursing staff should minimize handling neonates and do so with extra care when absolutely required. Further, measures should be taken to comfort the neonate before starting the procedure. Cortisol participates in human growth and metabolism. As an inhibitory feedback factor in traumatic stress response, a change in cortisol levels is not only the normal response of the human body to stress but also an objective index to measure the magnitude of the human stress response^16^. Salivary cortisol is measured as free cortisol. Blood-free cortisol diffuses into saliva and is unrelated to the saliva flow rate. Compared with blood cortisol, salivary cortisol can quickly and reliably reflect the change in plasma cortisol concentration^17^. Cortisol-binding globulin (CBG) has a high affinity for cortisol and plays a key role in studies related to Cushing’s syndrome and other diseases^18,19^. Studying CBG has the advantages of being noninvasive, safe, and repeatable. Currently, there are nearly 300 clinical direct and indirect nursing procedures. Because of the specific clinical need of ICU patients, the interval between routine procedures for patients cannot be more than 2 hours^20,21^. Previous studies have reported that the salivary cortisol reaction of premature infants was related to blood sampling and diaper changes, consistent with the results of this study^22,23^.

Meanwhile, the salivary cortisol levels in various night- and daytime procedures suggest that the nocturnal concentration of salivary cortisol in patients undergoing high-risk procedures changes significantly. The salivary cortisol of newborns has a circadian rhythm, which is usually high in the morning and low at night^24^. However, the findings of this study are not consistent with this trend, likely because the topic selected in this study is neonatal respiratory distress syndrome, which is related to the respiratory rate. The influence of diseases on cortisol secretion is mainly reflected in the change of pulse fluctuation amplitude based on circadian rhythm^25^. When the circadian rhythm of cortisol disappears, the fluctuation decreases. It has been reported that the circadian rhythm of cortisol disappears, and its fluctuation decreases in certain diseases such as chronic hypoxia, sleep apnea, and obesity. This condition is characterized as “passivation” of fluctuation^25^. Children secrete more growth hormones when sleeping. Stimulation and discomfort caused by procedures may affect their growth and development. Therefore, daily attention should be paid to the influence of nighttime procedures, and timely and predictable measures must be taken to reduce stress, relieve pain and strengthen pain management. Further, effective pain assessment is required for active pain interventions and pain management, essential to reduce the suffering of mechanically ventilated neonates.

## Conclusions

Saliva cortisol concentration index and heart rate can reflect the impact of routine operation stimulation on newborns and can, therefore, be monitored to manage neonatal pain.

## Data Availability

Data for the results of this study are available from the corresponding authors upon reasonable request.
